# Two new species of the genus *Trilacuna* from Chongqing, China (Araneae, Oonopidae)

**DOI:** 10.3897/zookeys.771.23158

**Published:** 2018-07-05

**Authors:** Yanfeng Tong, Xijin Guan, Shuqiang Li

**Affiliations:** 1 Life Science College, Shenyang Normal University, Shenyang 110034, China; 2 Institute of Zoology, Chinese Academy of Sciences, Beijing 100101, China

**Keywords:** Asia, copulatory organs, Gamasomorphinae, spider, taxonomy

## Abstract

Two new species of the genus *Trilacuna* Tong & Li, 2007, *T. simianshan* Tong & Li, sp. n. and *T. songyuae* Tong & Li, sp. n., are described from Simianshan Natural Reserve, Chongqing, China.

## Introduction

The spider genus *Trilacuna* was established by Tong and Li in 2007 to accommodate two new species, *T. angularis* Tong & Li, 2007 and *T. rastrum* Tong & Li, 2007, from Southwest China. Subsequently, additional species have been described from Thailand, Malaysia and Sumatra (Eichenberger and Kranz-Baltensperger 2011), Vietnam (Tong and Li 2013), the Himalayan region (Grismado et al. 2014), Iran (Malek-Hosseini et al. 2015), and Korea (Seo 2017). Currently, the genus *Trilacuna* comprises 20 species known from Asia (World Spider Catalog 2018).

This genus was originally diagnosed by the enlarged male palpal femora, the very complicated embolus-conductor complex, the branched endites in males and the notched labium (Tong and Li 2007). Grismado et al. (2014) re-diagnosed *Trilacuna* by the loss of the furrow connecting the posterior spiracles in males. However, as already discussed by Grismado et al. (2014) and Malek-Hosseini et al. (2015), some species, i.e., *T. aenobarba* (Brignoli, 1978), *T. bangla* Grismado & Ramírez, 2014, *T. hazara* Grismado & Ramírez, 2014 and *T. qarzi* Malek Hosseini & Grismado, 2015, have a shallow groove connecting the spiracles in males; *T. diabolica* Kranz-Baltensperger, 2011 and *T. werni* Eichenberger, 2011 have a well-developed furrow connecting the spiracles in males. So far, all known species of *Trilacuna* have a long postepigastric scutum in the females. This character is useful to distinguish *Trilacuna* from the other genera in the “*Dysderoides* complex” (Grismado et al. 2014; Tong and Li 2015).

In this paper two new *Trilacuna* species, *T. simianshan* Tong & Li, sp. n. and *T. songyuae* Tong & Li, sp. n., collected in the Simianshan Mountains, are described and illustrated.

## Materials and methods

The specimens were examined using a Leica M205C stereomicroscope. Details were studied under an Olympus BX51 compound microscope. Photos were made with a Canon EOS 550D zoom digital camera (18 megapixels) mounted on an Olympus BX51 compound microscope. Vulvae were cleared in lactic acid. For scanning electron microscopy (SEM), specimens were air-dried and uncoated. Pictures were taken with a Hitachi TM3030. All measurements were taken using an Olympus BX51 compound microscope and are in millimeters.

The following abbreviations are used in the text and figures:

**ALE** anterior lateral eyes;

**apo** apodemes;

**blo** broom-like outgrowth;

**boc** booklung covers;

**cll** cluster of long line-like structure;

**cp** circular projection;

**css** cone-shaped structure;

**dd** dark dot;

**dk** dark knob;

**ehb** elevated hair base;

**fo** fold;

**ldi** labium deep incision;

**mp** membranous projection;

**nls** numerous, long setae;

**ogr1** outgrowth 1;

**ogr2** outgrowth 2;

**pe** posterior extension;

**PLE** posterior lateral eyes;

**PME** posterior median eyes;

**psc** paddle-like sclerite;

**psp** posterior spiracles;

**rlo** ribbon-like outgrowth;

**rp** rectangular projection;

**sdb** slightly distal branch;

**slh** small hole;

**sls** slender line-like structure;

**sp** sperm pore;

**spr** small projection;

**sps** spear-like setae;

**sso** sector-shaped outgrowth;

**ssp** sickle-shaped protuberance;

**tss** two long, strong setae.

Type material is deposited in Shenyang Normal University (**SYNU**) and the Institute of Zoology, Chinese Academy of Sciences in Beijing (**IZCAS**).

## Taxonomy

### Trilacuna

Taxon classificationAnimaliaAraneaeOonopidae

Tong & Li, 2007

#### Type species.

*Trilacuna rastrum* Tong & Li, 2007.

### Trilacuna simianshan

Taxon classificationAnimaliaAraneaeOonopidae

Tong & Li sp. n.

http://zoobank.org/899E9966-88FD-4512-AE01-1C74A42ECB3F

**Figure 1.**
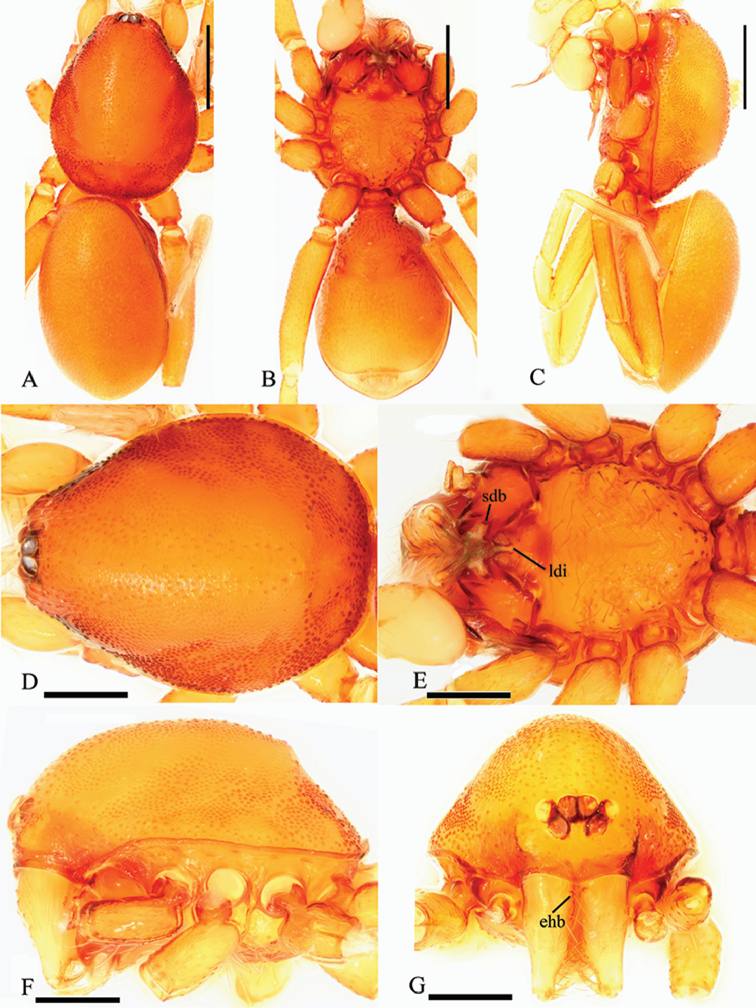
*Trilacuna simianshan* sp. n., male. **A** habitus, dorsal view **B** habitus, ventral view **C** habitus, lateral view **D** prosoma, dorsal view **E** prosoma, ventral view **F** prosoma, lateral view **G** prosoma, anterior view. Abbreviations: ehb = elevated hair base; ldi = labium deep incision; sdb = slightly distal branch. Scale bars: 0.2 mm (**A–C**); 0.1 mm (**D–G**).

**Figure 2.**
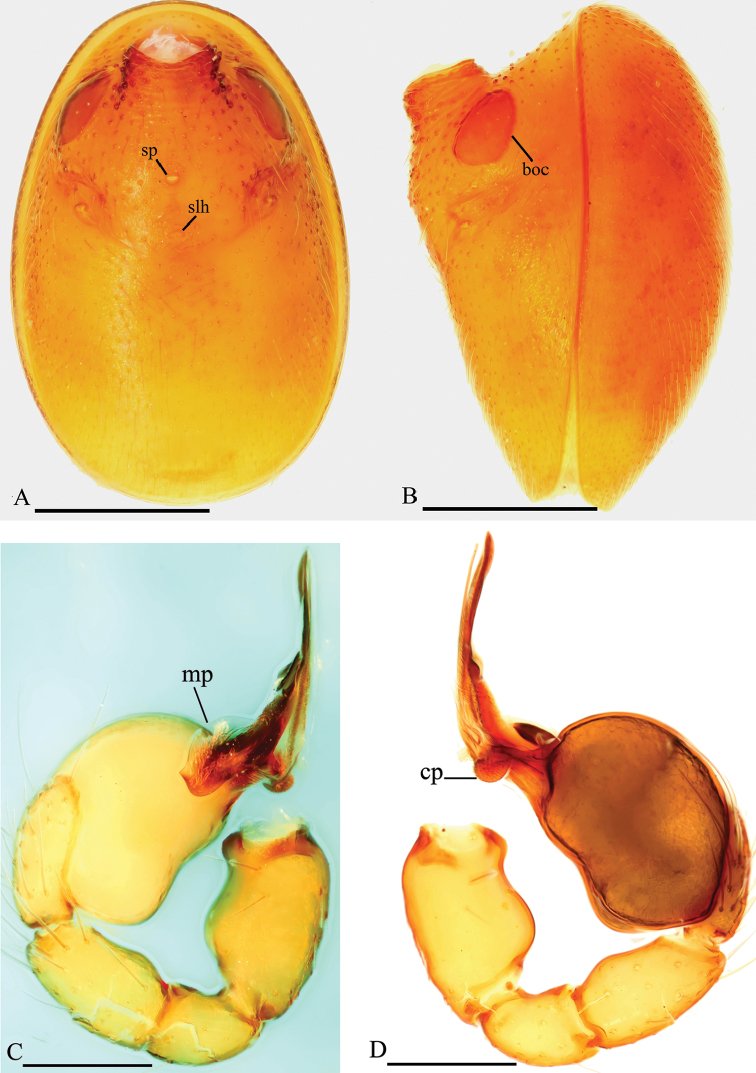
*Trilacuna simianshan* sp. n., male. **A** abdomen, ventral view **B** abdomen, lateral view **C** left palp, prolateral view **D** left palp, retrolateral view. Abbreviations: boc = booklung covers; cp = circular projection; mp = membranous projection; slh = small hole; sp = sperm pore. Scale bars: 0.2 mm.

**Figure 3.**
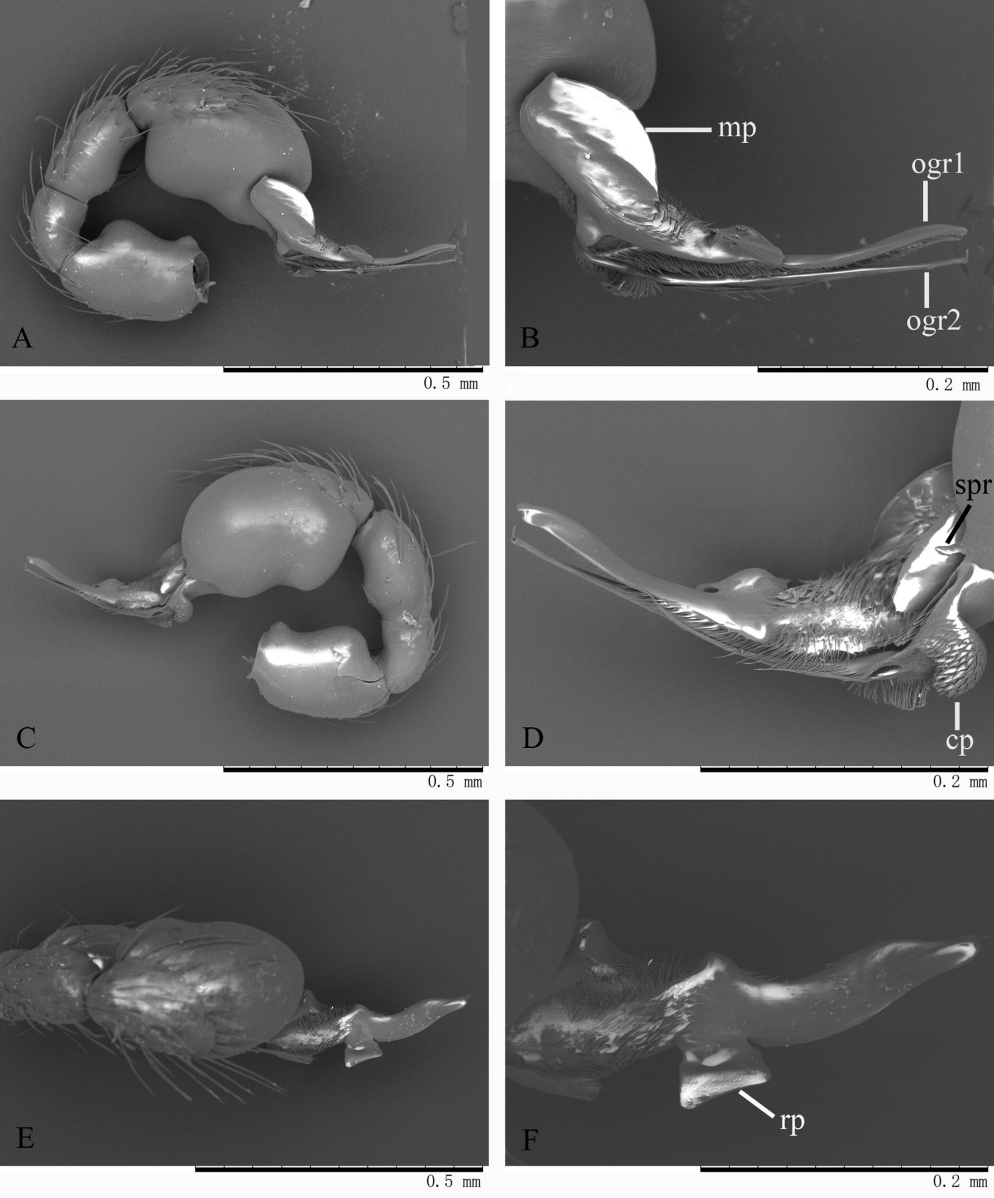
*Trilacuna simianshan* sp. n., male, SEM. **A** left palp, prolateral view **B** embolus system, prolateral view **C** left palp, retrolateral view **D** embolus system, retrolateral view **E** left palp, dorsal view **F** embolus system, dorsal view. Abbreviations: cp = circular projection; mp = membranous projection; ogr1 = outgrowth 1; ogr2 = outgrowth 2; rp = rectangular projection; spr = small projection.

**Figure 4.**
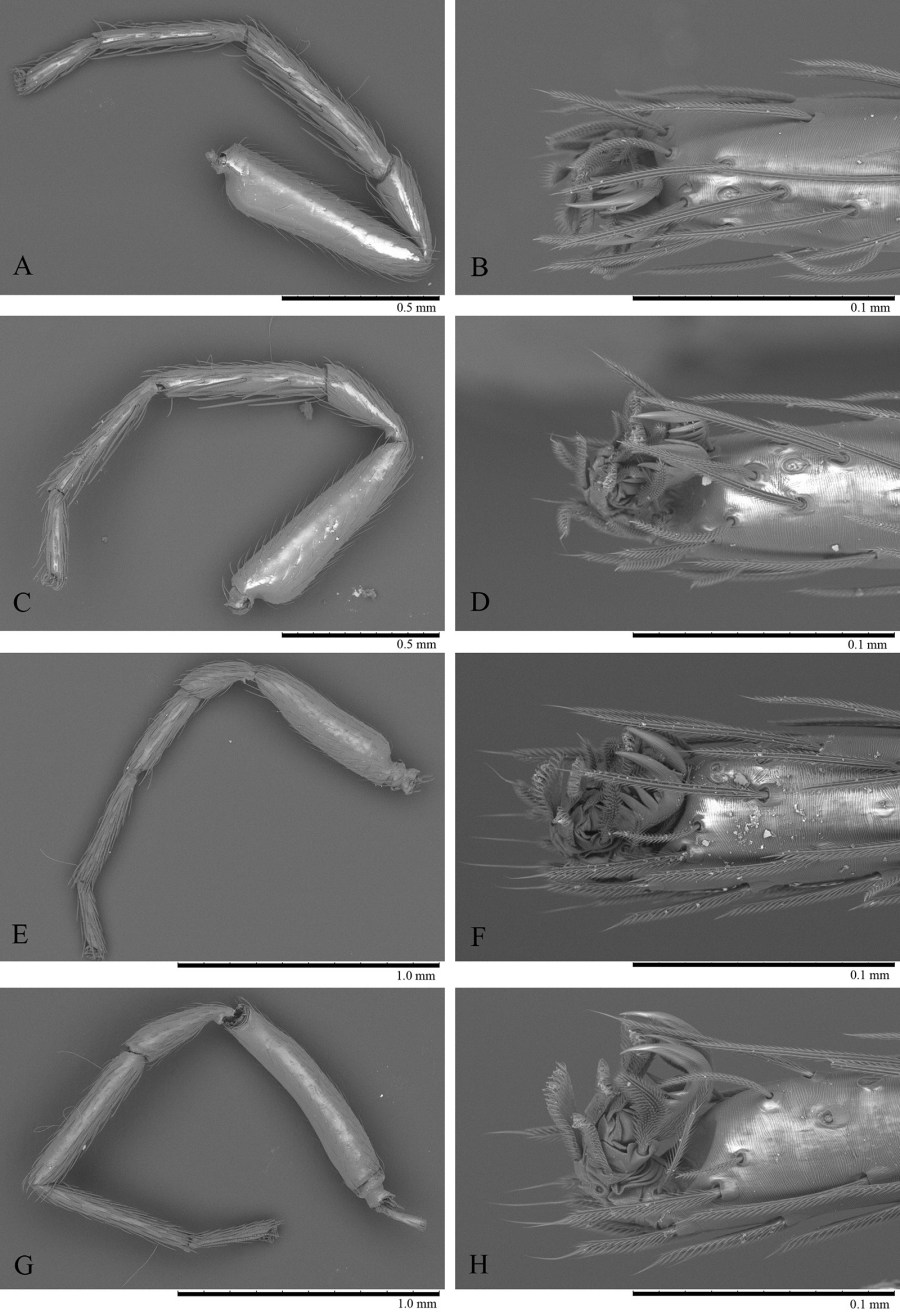
*Trilacuna simianshan* sp. n., female, right legs, SEM. **A** leg I, prolateral view **B** tarsus I, dorsal view **C** leg II, prolateral view **D** tarsus II, dorsal view **E** leg III, prolateral view **F** tarsus III, dorsal view **G** leg IV, prolateral view **H** tarsus IV, prolateral view.

**Figure 5.**
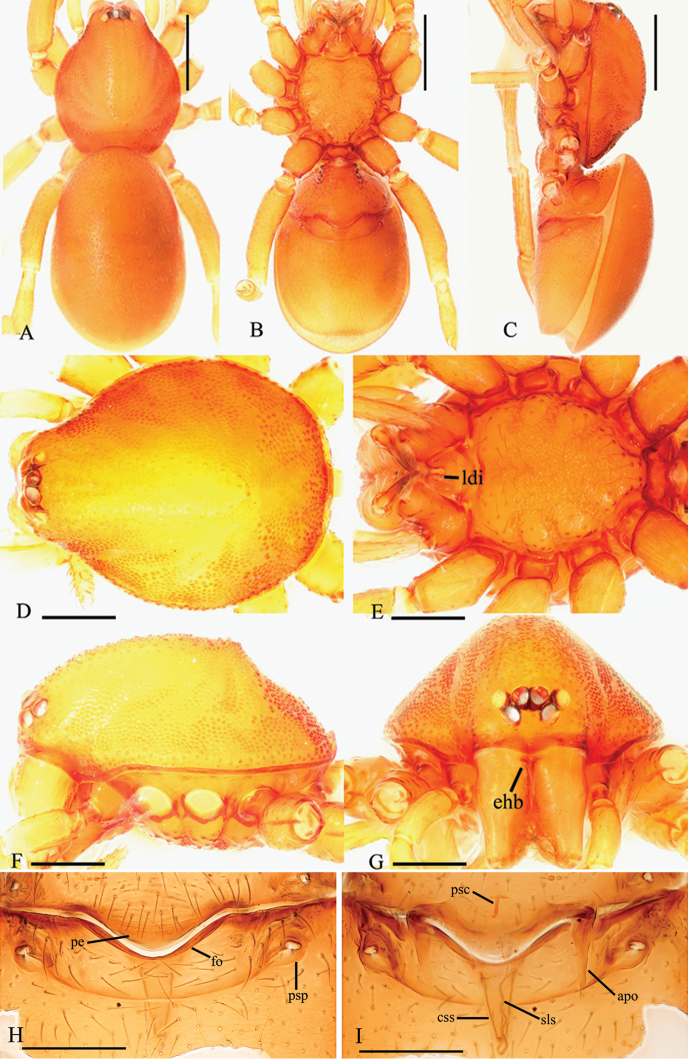
*Trilacuna simianshan* sp. n., female. **A, B, C** habitus, dorsal, ventral and lateral views **D, E, F, G** prosoma, dorsal, ventral, lateral and anterior views **H, I** genitalia, ventral and dorsal views. Abbreviations: apo = apodemes; css = cone-shaped structure; ehb = elevated hair base; fo = fold; ldi = labium deep incision; pe = posterior extension; psc = paddle-like sclerite; psp = posterior spiracles; sls = slender line-like structure. Scale bars: 0.2 mm (**A–C**); 0.1 mm (**D–I**).

Figs 1 , 2 , 3 , 4 , 5

#### Type material.

**Holotype** ♂ (SYNU-99), China, Chongqing Municipality, Jiangjin Dist., Simianshan Natural Reserve, Dawopu, 28°35'14.628"N, 106°22'44.790"E, 1000 m, 20.X.2014, leg. Y. Tong. **Paratypes**: 1 ♂, 2 ♀ (SYNU-99), same data as holotype; 1 ♂, 2 ♀ (IZCAS Ar-25089), same data as holotype; 2 ♀ (SYNU-100), China, Chongqing Municipality, Jiangjin Dist., Simianshan Natural Reserve, Dawopu, 28°34'43.956"N, 106°21'2.424"E, 28 m, 20.X.2014, leg. Y. Tong.

#### Etymology.

The specific name is a noun in apposition taken from the type locality.

#### Diagnosis.

This new species is similar to *T. rastrum* and can be distinguished by two long outgrowths of the embolus system and the long cone-shaped structure in females *vs.* the embolus system with a short ribbon-like outgrowth and a rake-shaped protuberance, and a simple stick-shaped sclerite centrally on the female genitalia of *T. rastrum* (see Tong and Li 2007: figs 6–10).

#### Description.

Male. Body yellow-brown, chelicerae and sternum lighter, legs yellow. Habitus as in Fig. 1A–C. Body length 2.21; carapace 1.13 long, 0.86 wide; abdomen 1.16 long, 0.75 wide. Carapace sides granulate; lateral margin rebordered, with a row of short, fine hairs and small blunt denticles. Six eyes, well developed, arranged in a compact group; ALE, PME subequal, larger than PLE; ALE–PLE separated by less than ALE radius, PME touching each other; posterior row recurved from above, procurved from front (Fig. 1D, G). Clypeus sinuous in frontal view, anterior lateral eyes separated from edge of carapace by about 2.0 times their diameter, with needle-like setae. Mouthparts: chelicerae straight, proximal region with one hair with elevated hair base (ehb); labium rectangular, anterior margin deeply incised (ldi) (Fig. 1E); endites slender, distally branched (sdb) (Fig. 1E). Sternum with radial furrows between coxae I–II, II–III, III–IV; surface strongly rugose on radial furrows and middle area; setae sparse, light, needle-like, evenly scattered (Fig. 1E). Abdomen: booklung covers large, ovoid, surface smooth (Fig. 2B). Dorsal scutum not fused to epigastric scutum. Apodemes present, posterior spiracles connected by a shallow groove (Fig. 2A). Leg spination (all spines longer than segment width): legs I-II: tibia: v2-2-2-2-0, metatarsus: v2-2-0. Trichobothria: tibia: each with three; metatarsus: each with one.

Genitalia. Epigastric region with sperm pore (sp) small, oval, rebordered, situated between anterior spiracles; with a small hole (slh) between the posterior spiracles (Fig. 2A). Palp (Figs 2C, D, 3): orange. 0.46 long (0.15, 0.08, 0.11, 0.12). Femur strongly swollen (width/length = 0.09/0.15) (Fig. 2C, D). Bulb oval, stout, tapering apically. Embolus system (Fig. 3B, D, F) complex, with two long, strongly curved outgrowths (ogr1 and ogr2); the surface of the embolus system bearing numerous small “papillae”. The base of the embolus system with a wing-like, membranous projection (mp) in prolateral view and a circular projection (cp) covered with scales in retrolateral view; middle part of the embolus system with a rectangular projection (rp) in dorsal view.

Female. As in male except as noted. Slightly larger than male. Body length 2.28; carapace 0.99 long, 0.85 wide; abdomen 1.36 long, 0.89 wide. Postepigastric scutum long. Posterior spiracles connected by groove (Fig. 5B).

Female genitalia. Ventral view (Fig. 5H): Middle part of posterior margin of epigastric scutum much extended posteriorly (pe); surface without external features. Dorsal view (Fig. 5I): with a very long, nearly cone-shaped structure (css), at the posterior end of the cone-shaped structure is a slender line-like structure (sls) originating and extending anteriorly. Transverse bars with two relatively long, lateral apodemes.

#### Distribution.

China (Chongqing).

### Trilacuna songyuae

Taxon classificationAnimaliaAraneaeOonopidae

Tong & Li sp. n.

http://zoobank.org/737A9BE2-8B18-4FA6-AB7E-D31F25495E0F

**Figure 6.**
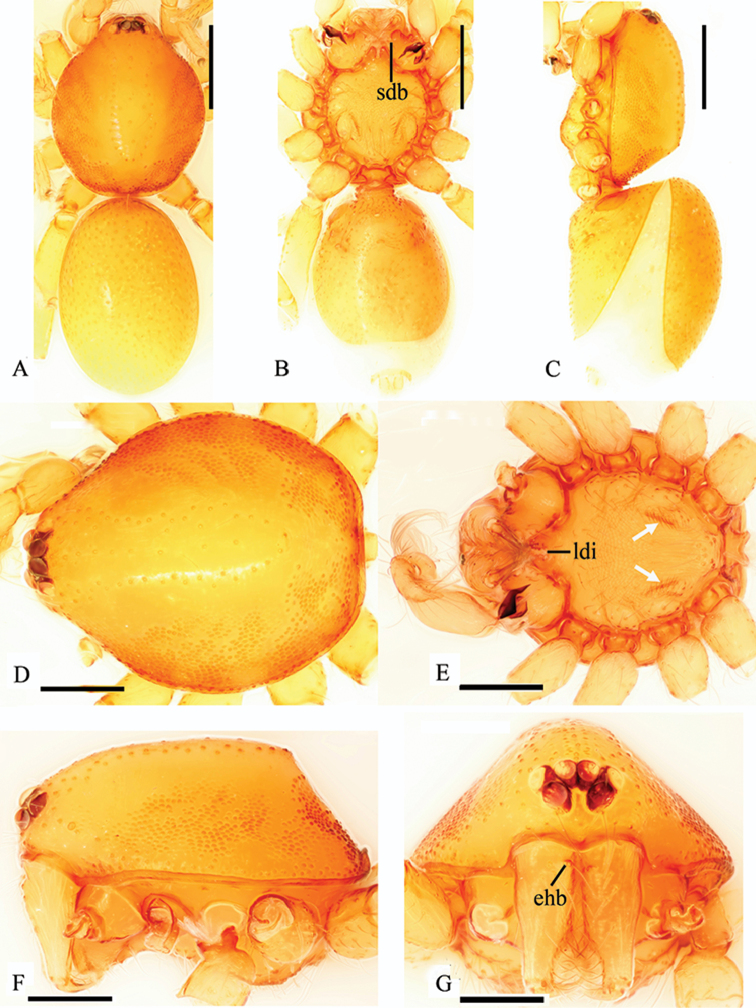
*Trilacuna songyuae* sp. n., male. **A** habitus, dorsal view **B** habitus, ventral view **C** habitus, lateral view **D** prosoma, dorsal view **E** prosoma, ventral view, white arrow shows the ridges, with a row of setae **F** prosoma, lateral view **G** prosoma, anterior view. Abbreviations: ehb = elevated hair base; ldi = labium deep incision; sdb = slightly distal branch. Scale bars: 0.2 mm (**A–C**); 0.1 mm (**D–G**).

**Figure 7.**
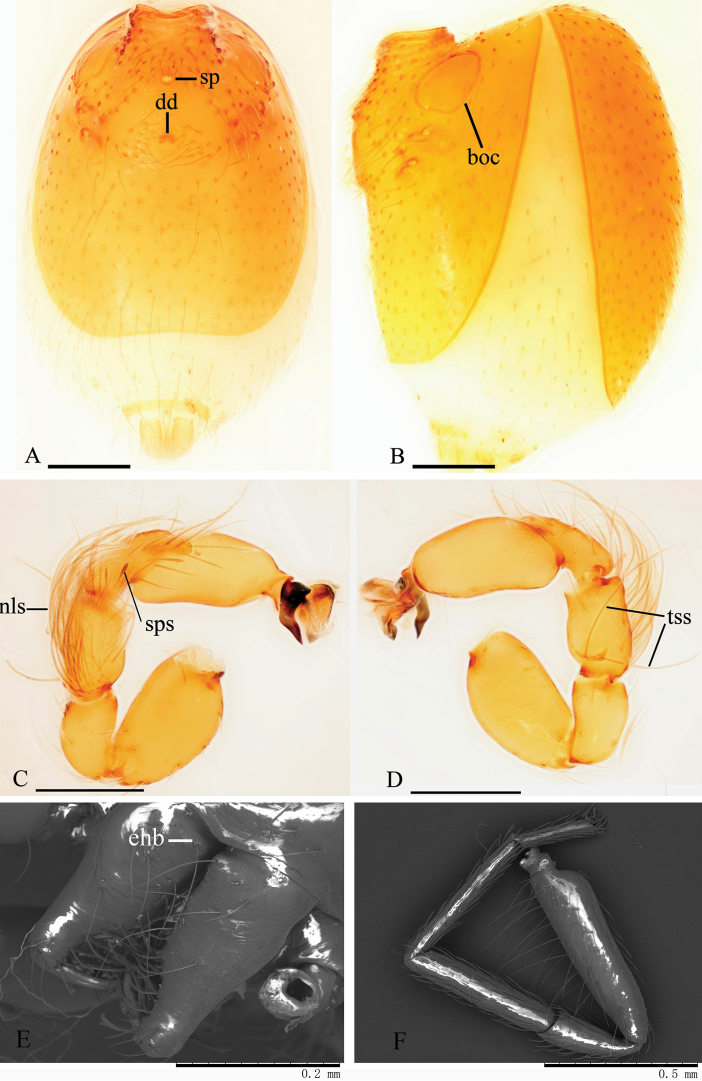
*Trilacuna songyuae* sp. n., male. **A** abdomen, ventral view **B** abdomen, lateral view **C** left palp, prolateral view **D** left palp, retrolateral view **E** chelicerae, oblique, anterior view **F** left leg IV, prolateral view. Abbreviations: boc = booklung covers; dd = dark dot; ehb = elevated hair base; nls = numerous, long setae; sp = sperm pore; sps = spear-like setae; tss = two long, strong setae. Scale bars: 0.1 mm (**A–D**).

**Figure 8.**
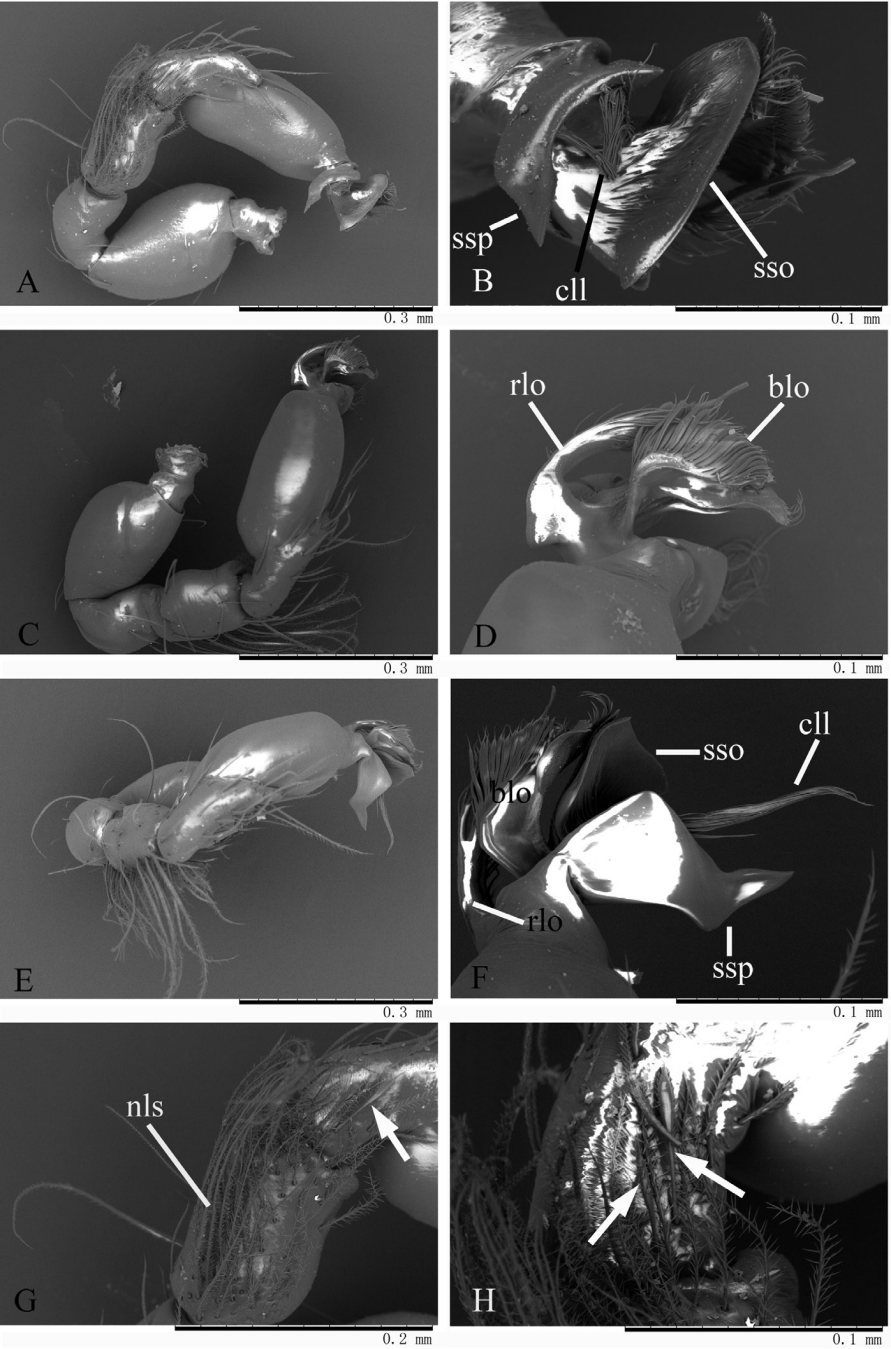
*Trilacuna songyuae* sp. n., male, SEM. **A** left palp, prolateral view **B** embolus system, prolateral view **C** left palp, retrolateral view **D** embolus system, retrolateral view **E** left palp, dorsal view **F** embolus system, dorsal view **G** tibia and cymbium, prolateral view **H** same, details, white arrow shows the spear-like setae. Abbreviations: blo = broom-like outgrowth; cll = cluster of long line-like structure; nls = numerous, long setae; rlo = ribbon-like outgrowth; sso = sector-shaped outgrowth; ssp = sickle-shaped protuberance.

**Figure 9.**
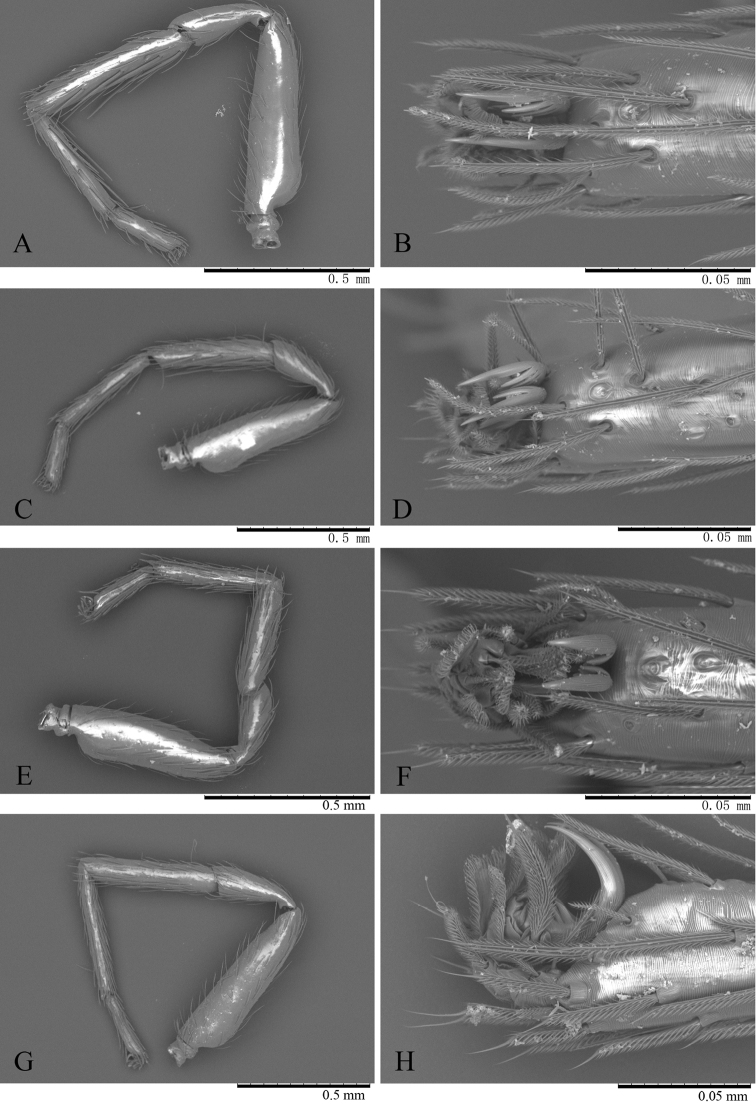
*Trilacuna songyuae* sp. n., female, right legs, SEM. **A** leg I, prolateral view **B** tarsus I, dorsal view **C** leg II, prolateral view **D** tarsus II, dorsal view **E** leg III, prolateral view **F** tarsus III, dorsal view **G** leg IV, prolateral view **H** tarsus IV, prolateral view.

**Figure 10.**
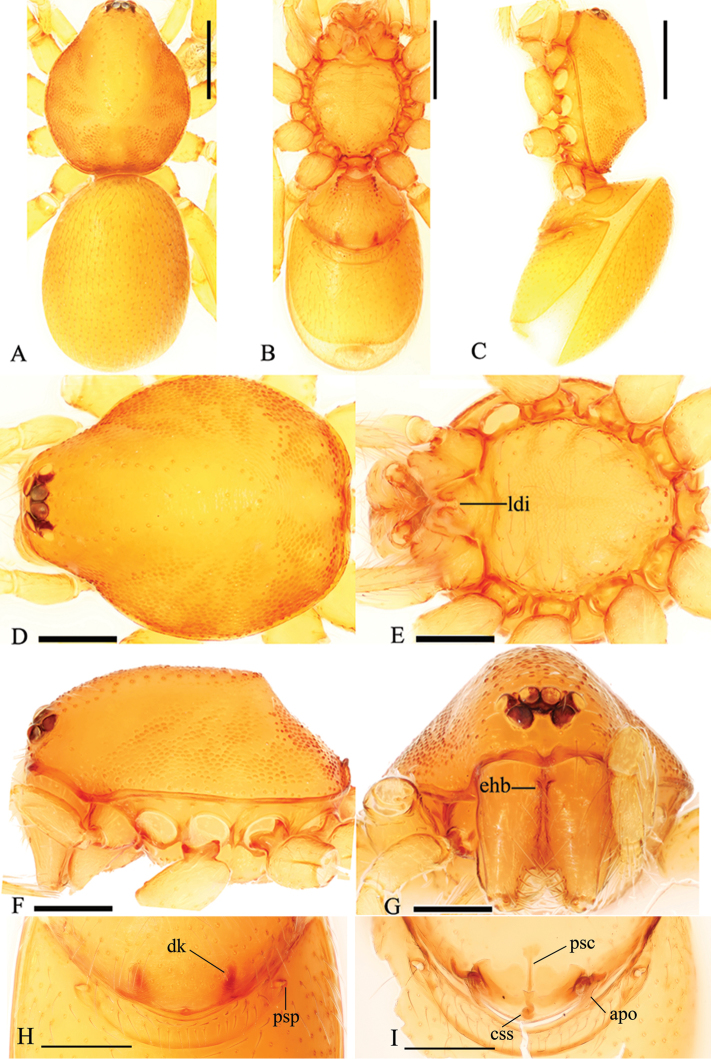
*Trilacuna songyuae* sp. n., female. **A, B, C** habitus, dorsal, ventral and lateral views **D, E, F, G** prosoma, dorsal, ventral, lateral and anterior views **H, I** genitalia, ventral and dorsal views. Abbreviations: apo = apodemes; css = cone-shaped structure; dk = dark knob; ehb = elevated hair base; ldi = labium deep incision; psc = paddle-like sclerite; psp = posterior spiracles. Scale bars: 0.2 mm (**A–C**); 0.1 mm (**D–I**).

Figs 6 , 7 , 8 , 9 , 10

#### Type material.

**Holotype** ♂, (SYNU-101), China, Chongqing Municipality, Jiangjin Dist., Simianshan Natural Reserve, Dawopu, 28°34'43.956"N, 106°21'2.424"E, 28 m, 20.X.2014, leg. S. Lyu and Y. Tong. **Paratypes**: 2 ♂, 2 ♀ (SYNU-101), same data as holotype; 5 ♂, 4 ♀ (SYNU-102), same data as holotype; 7 ♂, 5 ♀ (IZCAS Ar-25088), China, Chongqing Municipality, Jiangjin Dist., Simianshan Natural Reserve, Dawopu, 28°35'14.628"N, 106°22'44.790"E, 1000 m, 20.X.2014, leg. S. Lyu and Y. Tong.

#### Other material studied.

7 ♂, 1 ♀ (SYNU-103), same data as holotype; 8 ♂, 2 ♀ (SYNU-105), China, Chongqing Municipality, Jiangjin Dist., Simianshan Natural Reserve, Dawopu, 28°35'14.628"N, 106°21'44.790"E, 1000 m, 20. X. 2014, leg. S. Lyu and Y. Tong.

#### Etymology.

The specific name is after Miss Songyu Lyu (吕松宇), one of the collectors of this species.

#### Diagnosis.

The new species is similar to *T. hansanensis* Seo, 2017. Both species have an elevated ridge on the posterior part of the male sternum, but can be distinguished by the long oval bulb, the very long setae (nls) on the prolateral surface of the male palpal tibiae and the two small, spear-like setae (sps) on the basal part of the prolateral surface of male palpal cymbium. *Trilacuna hansanensis* has a pear-shaped bulbus, and there are no special setae on the male palpal tibia or cymbium (see Seo 2017: figs 1A–K).

#### Description.

Male. Body yellow-brown, chelicerae and sternum lighter, legs yellow. Habitus as in Fig. 6A–C. Body length 1.87; carapace 0.86 long, 0.73 wide; abdomen 0.97 long, 0.71 wide. Carapace sides granulate; lateral margin rebordered, with a row of short, fine hairs and small, blunt denticles. Eyes six, well developed, arranged in a compact group; ALE largest, PLE smallest; ALE–PLE separated by less than ALE radius, PME touching each other; posterior row recurved from above, procurved from front (Fig. 6D, G). Clypeus sinuous in frontal view, anterior lateral eyes separated from edge of carapace by about 2.0 times their diameter, with needle-like setae. Mouthparts: chelicerae straight, proximal region with one hair with elevated hair base (ehb); labium rectangular, anterior margin deeply incised (ldi) (Fig. 6E); endites slender, distally slightly branched (sdb) (Fig. 6B). Sternum with radial furrows between coxae I–II, II–III, III–IV; lateral margin smooth, middle area reticulate, posterior part with two slightly elevated ridges, each covered with a row of short, strong setae, the region between the two ridges strongly wrinkled (Fig. 6E). Abdomen: booklung covers (boc) large, ovoid, surface smooth (Fig. 7B). Dorsal scutum not fused to epigastric scutum. Apodemes present, posterior spiracles not connected by groove (Fig. 7A). Leg spination (all spines longer than segment width): legs I‒II: tibiae: v2-2-2-2-0, metatarsi: v2-2-0, leg IV: femur with very long hairs ventrally (Fig. 7F). Trichobothria: tibia: each with 3; metatarsus: each with 1.

Genitalia. Epigastric region (Fig. 7A) with sperm pore small, oval, rebordered, situated before anterior spiracles; with a small dark dot (dd) between the posterior spiracles, a cluster of long hairs around the dark dot. Palp (Figs 7C, D, 8): orange. 0.42 long (0.14, 0.07, 0.10, 0.11). Femur strongly swollen (width/length = 0.08/0.14) (Fig. 7C, D). Tibia with numerous, very long, penniform setae (nls) on prolateral surface and two long, strong setae (tss) on retrolateral surface (Fig. 7C, D). Cymbium with two small, spear-like setae (sps) on basal part of prolateral surface (Figs 7C, 8G, H). Bulb long, oval, stout, tapering apically. Embolus system (Fig. 8B, D, F) complex, bearing numerous small “papillae”; with a strongly sclerotized, sickle-shaped protuberance (ssp) and a fan-shaped outgrowth (sso) prolaterally; between the two outgrowths is a cluster of long, line-like structures (cll); with a ribbon-like, nearly transparent outgrowth (rlo) and a broom-like outgrowth (blo) retrolaterally.

Female. As in male except as noted. Slightly larger than male. Body length 1.91; carapace 0.89 long, 0.75 wide; abdomen 1.02 long, 0.78 wide. Postepigastric scutum long. Posterior spiracles connected by groove (Fig. 10B).

Genitalia. Ventral view (Fig. 10H): surface without external features, a dark knob-like marking (dk) can be seen through the cuticle. Dorsal view (Fig. 10I): with a very small, cone-shaped structure (css). Transverse bars with two relatively long, lateral apodemes.

#### Distribution.

China (Chongqing).

## Supplementary Material

XML Treatment for Trilacuna

XML Treatment for Trilacuna simianshan

XML Treatment for Trilacuna songyuae
